# Efficacy of Phase I and Phase II *Coxiella burnetii* Bacterin Vaccines in a Pregnant Ewe Challenge Model

**DOI:** 10.3390/vaccines11030511

**Published:** 2023-02-22

**Authors:** Sarah E. Williams-Macdonald, Mairi Mitchell, David Frew, Javier Palarea-Albaladejo, David Ewing, William T. Golde, David Longbottom, Alasdair J. Nisbet, Morag Livingstone, Clare M. Hamilton, Stephen F. Fitzgerald, Søren Buus, Emil Bach, Annemieke Dinkla, Hendrik-Jan Roest, Ad P. Koets, Tom N. McNeilly

**Affiliations:** 1 Moredun Research Institute, Pentlands Science Park, Bush Loan, Penicuik, Midlothian EH26 0PZ, UK; 2 Biomathematics and Statistics Scotland, JCMB, The King’s Buildings, Peter Guthrie Tait Road, Edinburgh EH9 3FD, UK; 3 Department of Immunology & Microbiology, Faculty of Health and Medical Sciences, University of Copenhagen, Blegdamsvej 3B, DK 2200 Copenhagen, Denmark; 4 Department of Bacteriology, Host-Pathogen Interaction and Diagnostics, Wageningen Bioveterinary Research, Houtribweg 39, 8221 RA Lelystad, The Netherlands; 5 Department of Population Health Sciences, Faculty of Veterinary Medicine, Utrecht University, Yalelaan 7, 3584 CL Utrecht, The Netherlands

**Keywords:** Q-fever, *Coxiella burnetii*, vaccine, sheep, phase I, phase II

## Abstract

The bacterium *Coxiella burnetii* can cause the disease Q-fever in a wide range of animal hosts. Ruminants, including sheep, are thought to play a pivotal role in the transmission of *C. burnetii* to humans; however, the only existing livestock vaccine, namely, Coxevac^®^ (Ceva Animal Health Ltd., Libourne, France), a killed bacterin vaccine based on phase I *C. burnetii* strain Nine-Mile, is only approved for use in goats and cattle. In this study, a pregnant ewe challenge model was used to determine the protective effects of Coxevac^®^ and an experimental bacterin vaccine based on phase II *C. burnetii* against *C. burnetii* challenge. Prior to mating, ewes (*n* = 20 per group) were vaccinated subcutaneously with either Coxevac^®^, the phase II vaccine, or were unvaccinated. A subset of pregnant ewes (*n* = 6) from each group was then challenged 151 days later (~100 days of gestation) with 10^6^ infectious mouse doses of *C. burnetii*, Nine-Mile strain RSA493. Both vaccines provided protection against *C. burnetii* challenge as measured by reductions in bacterial shedding in faeces, milk and vaginal mucus, and reduced abnormal pregnancies, compared to unvaccinated controls. This work highlights that the phase I vaccine Coxevac^®^ can protect ewes against *C. burnetii* infection. Furthermore, the phase II vaccine provided comparable levels of protection and may offer a safer and cost-effective alternative to the currently licensed vaccine.

## 1. Introduction

*Coxiella burnetii* (*C. burnetii*) is a highly infectious obligate intracellular bacterium that is the etiologic agent of the zoonosis Q-fever. The disease was first identified in abattoir workers in Australia as a febrile illness of unknown origin termed query fever (Q-fever) [1]. The bacterium has a pan-global distribution with the exception of New Zealand and Antarctica [2,3].

A wide range of animals, as well as humans, can be infected with *C. burnetii*; however, domestic ruminants, principally dairy cows, sheep, and goats, are the main reservoirs and are responsible for most Q-fever outbreaks [4,5,6]. Infected livestock generally lack clinical signs prior to the onset of adverse reproductive outcomes, including the birth of weak offspring, abortion and infertility [7]. Clinical disease is more commonly observed in small ruminants with symptoms rarely described in cattle [8]. Ruminants can shed *C. burnetii* via multiple routes, including milk, faeces, vaginal mucus, and birthing products [9,10]. During normal parturition or abortive episodes, exceptionally high numbers of bacteria can be shed into the environment, and typically, infection ensues via the inhalation of contaminated aerosols, which can travel up to 18 km [11,12]. 

In humans, the non-specific clinical symptoms associated with *C. burnetii* infection mean it is seldom diagnosed. The disease manifestations of Q-fever range from acute flu-like symptoms to persistent focalized infections (formerly referred to as ‘chronic Q-fever’), which can be life limiting and/or threatening, including endocarditis and hepatitis [13]. During pregnancy, Q-fever can cause perinatal complications, including miscarriage, preterm delivery, or low birth weights in humans [14]. The largest outbreak ever recorded occurred in the Netherlands between 2007 and 2011, with over 4000 human cases reported. The principal source of this outbreak was identified as dairy goats and to a smaller extent sheep [15]; however, it should be noted that sheep are responsible for a large proportion of human Q-fever outbreaks [7]. Throughout the world and particularly in many African countries, ruminant production plays a crucial role in ensuring food security. The socio-economic consequences of Q-fever, due to the impact on human health, associated expenditure, and reductions in livestock productivity, are thought to be vastly underappreciated [16]. The total cost of the Netherlands outbreak (2007–2011) was estimated at over EUR 300 million [17]. Furthermore, by 2050, the global goat and sheep population is estimated to increase by 60%, to 2.7 billion, emphasizing the need to improve and expand current Q-fever control measures [18]. 

Evolution of *C. burnetii* is thought to occur via clonal expansion with limited genetic variability between isolates from the same genomic group, of which six have been proposed [19]. Recently, Hemsley et al. [20] have shown that point mutations can cause significant variability between genomic groups, affecting protein expression and may generate antigen profiles which differ between groups. These mutations may facilitate disparate interactions between *C. burnetii* isolates and the host immune system and need to be considered during the development of control strategies. Antigenic phase variation occurs in *C. burnetii* upon serial passage, in cell culture or in embryonated hen’s eggs, from a virulent phase I form to the avirulent phase II form. Phase I *C. burnetii*, isolated from natural sources, contain a full-length lipopolysaccharide (LPS), while phase II *C. burnetii* possess a truncated LPS. Additionally, a third phenotypic mutant, termed Nine-Mile Crazy (NMC) (RSA514), has attenuated virulence, and contains LPS of intermediate length between phase I and phase II [21,22]. The shortened LPS form contains a lipid A membrane anchored to an inner core but lacks the outer core and repeating O-antigen sugars, virenose and dihydrohydroxystreptose, present in phase I variants [23]. Previously, LPS truncation was attributed solely to a 26 kb and 31.5 kb chromosomal deletion in phase II and NMC, respectively, which eliminated several open reading frames responsible for biosynthesis of O-antigen sugars [24,25]. However, recently, whole-genome sequencing studies have shown that phase variation can occur via multiple genetic mutation mechanisms [26]. 

Due to the ubiquitous nature of *C. burnetii*, vaccines are considered the most effective way to control transmission. Currently, there are two commercially available Q-fever vaccine formulations, Q-VAX^®^ (Commonwealth Serum Laboratories Ltd., Melbourne, Australia) and Coxevac^®^ (Ceva Animal Health Ltd.), both produced using formalin inactivated phase I *C. burnetii* antigens. Q-VAX^®^, a human Q-fever vaccine, is solely licensed for use in Australia and requires pre-screening due to the potential for adverse reactogenicity in individuals with prior *C. burnetii* exposure [27]. Coxevac^®^ is a livestock vaccine licensed for use in cattle and goats and is not currently approved for use in sheep [28]. Despite generating high levels of protection, the safety of phase I vaccines is a major issue considering that severe local and systemic reactions can occur post vaccination; it has been documented that Coxevac^®^ can cause painful injection site reactions, an increase in body temperatures and a decrease in milk yields [29]. Additionally, the manufacture of phase I vaccines involves the culture of *C. burnetii* at containment level 3 (CL3), which has both cost and human safety issues and has limited the deployment of these vaccines [30]. Vaccines based on phase II antigens can be produced at a lower containment level, CL2, reducing both associated risk and cost; however, these have been shown to be non-protective in goats and guinea pigs [31,32]. Few studies have assessed the impact of phase I vaccines on sheep, and no studies have investigated the protective potential of phase II vaccines in pregnant ewes [33].

The aim of the study presented here was to investigate the efficacy of a phase I vaccine and a phase II vaccine in pregnant ewes experimentally challenged with *C. burnetii*. Protection was determined by reductions in adverse pregnancy outcomes, shedding of *C. burnetii* in milk, faeces and vaginal secretions and tissue levels of bacteria. Vaccines used in this study were the commercially available phase I *C. burnetii* vaccine, Coxevac^®^ (strain RSA 493/Nine-Mile phase I), and a non-commercial vaccine formulation composed of *C. burnetii* phase II antigens adjuvanted with Quil-A.

## 2. Materials and Methods

### 2.1. Propagation of C. burnetii Strain

*Coxiella burnetii* Nine-Mile strain RSA493 was cultured in Buffalo Green Monkey (BGM) cells by colleagues at Wageningen Bioveterinary Research, the Netherlands, as described previously [34]. This strain was originally isolated in Montana, in 1935, from the tick *Dermacentor andersoni* [35]. Prior to inoculation the concentration of the strain was adjusted to 10^6^ infective mouse doses (IMD)/mL. Aliquots were stored at −70 °C until use.

### 2.2. Experimental Design

#### 2.2.1. Animals

Adult female 3-year-old Texel-cross ewes, which had previously been vaccinated against *Chlamydia abortus* (*C. abortus*), were used in this study. Ewes were initially kept on pasture and then moved to the Moredun High Security Unit (HSU) prior to *C. burnetii* challenge. In the HSU, all study animals had access to water ad libitum and were fed appropriate volumes of Premium 18 sheep nuts (Harbro, UK) and ad libitum hay. Energise feed mineral lick tubs (Harbro, UK) were provided during the last two weeks of pregnancy. Prior to the start of the study, all ewes were negative for *C. burnetii* antibodies as determined by enzyme-linked immunosorbent assay (ELISA) detailed in Section 2.5.2.

#### 2.2.2. Immunization and Challenge

**Figure 1:**
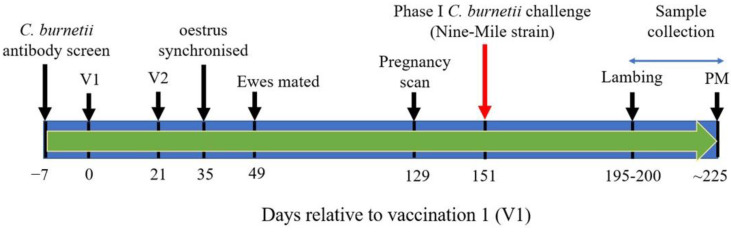
Immunization and *C. burnetii* challenge schedule. V1 and V2 = vaccination 1 and 2, respectively; PM = post-mortem.

The experimental protocol for the immunization study is summarized in Figure 1. Ewes were randomly allocated into three groups and were immunized via the subcutaneous route on two occasions three weeks apart with either 2 mL Coxevac^®^ (Lot. 0807FG1A, Ceva Animal Health Ltd., Libourne, France) containing formaldehyde inactivated phase I *C. burnetii* Nine-Mile strain RSA493 plus thiomersal preservative in PBS (Group 1, *n* = 20), or 2 mL of formalin inactivated phase II *C. burnetii* Nine-Mile strain (Lot. SAI.CH, Virion/Serion, Würzburg, Germany) containing 1 mg Quil-A (Brenntag Biosector, Frederikssund, Denmark) and thiomersal preservative in PBS (Group 2, *n* = 20). Phase II *C. burnetii* was generated following 166 passages in embryonated hen eggs (personal communication, Bioscience Slovakia, Bratislava, Slovakia). To ensure the phase I and phase II bacterin vaccines contained equivalent numbers of *C. burneti*, quantitative PCR (qPCR) using the Adiavet™ *Coxiella* Real Time kit (Bio-X Diagnostics, Rochefort, Belgium) was carried out following the manufacturer’s instructions. Both vaccines were adjusted to 1.20 × 10^10^ genome copy equivalents per dose. A third group of ewes acted as unvaccinated controls (Group 3, *n* = 20). 

Two weeks after the second immunization, oestrus was synchronized in ewes using Chronogest CR 20 mg controlled flugestone acetate release vaginal sponges (MSD Animal Health, Milton Keynes, UK), and ewes were mated two weeks later. A further 11 weeks later, ewes were ultrasound scanned to determine pregnancy status and six ewes from each of Groups 1–3, weighted for parity, were selected for *C. burnetii* challenge. Each group contained five twin-bearing ewes and one triplet-bearing ewe. At day 102 post mating, these ewes were challenged in the Moredun HSU with 10^6^ IMD of *C. burnetii* Nine-Mile strain RSA493 via the subcutaneous route.

#### 2.2.3. Assessment of Post-Vaccination Reactogenicity

The rectal temperatures of all ewes were taken immediately prior to each of the two vaccinations and then again at 24 h post vaccination, when animals were also inspected by a veterinarian for the presence of injection site reactions. 

#### 2.2.4. Sample Collection

Blood samples were collected from all ewes for serological analysis on a weekly basis for the first eight weeks after the first immunization, then every second week until pregnancy scanning. Ewes and surviving lambs were euthanized by overdose of sodium pentobarbitone at 32 weeks relative to the first immunization (~4 weeks post lambing) for post-mortem analysis. For challenged ewes only, blood samples were also collected weekly from the time of challenge until post-mortem. Milk, faeces and vaginal swab samples were collected from challenged ewes on days 0, 1, 2 and 3, relative to lambing/abortion, and weekly thereafter until post-mortem, for *C. burnetii* detection by qPCR. Placental samples, including at least one cotyledon and one inter-cotyledonary membrane, were collected at lambing/abortion. To determine the tissue distribution of *C. burnetii* in lambs and ewes, samples of spleen and liver were collected from aborted foetuses, stillborn lambs and lambs sacrificed at the end of the study. Samples of liver, spleen, uterine body, udder, supra-mammary lymph node (SMLN) and mammary gland were collected from sacrificed ewes. 

### 2.3. Preparation of Clinical Samples

Following collection, blood samples were left to clot overnight at room temperature. Samples were centrifuged for ten minutes at 2000× *g*. Serum was removed using a sterile pastette into a labelled 2 mL screw cap tube (Sarstedt, Nümbrecht, Germany). All samples, including tissue samples, were stored at −70 °C for future downstream processing and analyses.

### 2.4. DNA Extraction

DNA from approximately 200 mg of each faecal sample was extracted using a QIAamp DNA stool kit (Qiagen, Hilden, Germany) following the manufacturer’s instructions. DNA was extracted from milk, vaginal swab, and tissue samples using a DNeasy Blood and Tissue Kit (Qiagen, Germany), with any modifications outlined below. Proteinase K (20 µL) and buffer ATL (180 µL) was added to 200 µL milk samples, which were heated at 56 °C for one hour, after which the standard protocol was resumed. Vaginal swabs were added to 1.5 mL screw cap Eppendorf tubes containing 400 µL buffer ATL and 20 µL Proteinase K. Samples were vortexed and lysed at 56 °C for 10 min, and then 400 µL of buffer AL was then added to samples, after which the standard protocol was resumed. All tissue samples were lysed overnight at 56 °C. An extraction control, comprising 200 µL of sterile distilled water, was included in every run. To elute DNA, 50 µL of buffer AE (Qiagen, Germany) was used for all samples. Samples were stored at −70 °C prior to analysis.

### 2.5. Detection of Abortifacient Agents

To determine if abnormal lambing outcomes were attributable to *C. burnetii*, qPCR and ELISA assays targeting *C. burnetii*, *C. abortus* and *Toxoplasma gondii* (*T. gondii*), common ovine abortifacient agents, were carried out on a defined subset of samples:

#### 2.5.1. qPCR Detection of Abortifacient Agents

To detect the presence of *C. burnetii* DNA, all samples were subject to qPCR using an assay which targeted the multi-copy-number insertion sequence IS1111 [36,37]. To detect *C. abortus* DNA all placenta samples (cotyledon and inter-cotyledonary membrane) and vaginal swabs collected on day 0 post lambing were subject to qPCR, targeting the major outer membrane protein (MOMP) of *C. abortus* [38]. To detect *T. gondii* DNA, qPCR on a 529bp repeat element was carried out on all placenta samples (cotyledon and inter-cotyledonary membrane) and on lamb liver and spleen samples [39]. Primers and probes used in this study are detailed in Table 1. Briefly, the final 20 µL reaction volume for each qPCR consisted of 10 µL QuantiNova master-mix (Qiagen, Germany), the required concentrations of each forward and reverse primers and probes as stated in Table 1, 1x QN ROX™ reference dye (Qiagen, Germany), RNase and DNase free water (Invitrogen, Paisley, UK) and 3 or 5 µL of extracted DNA sample. For *C. burnetii* and *T. gondii* 5 µL of extracted DNA was used, while 3 µL was used for *C. abortus*. Two negative controls were included in each qPCR run: (i) a no-template control consisting of molecular grade water; (ii) the elution from the negative extraction controls. For *T. gondii* qPCRs, 0.01 fg competitive internal amplification control (CIAC) was also included in each PCR reaction. For *C. burnetii* qPCRs, a positive control sample consisting of genomic DNA prepared from *C. burnetii* Nine-Mile strain RSA493 was included in each PCR run. DNA was amplified using an ABI Prism^®^ 7000 Sequence Detection System (Thermo Fisher Scientific, Waltham, USA) in duplicate, for every sample, using the following conditions: *C. burnetii* qPCR: 1 cycle at 95 °C for 120 s followed by 45 cycles of 95 °C for 5 s and 60 °C for 5 s; *C. abortus* qPCR: 1 cycle at 95 °C for 10 min followed by 45 cycles of 95 °C for 15 s and 60 °C for 1 min; *T. gondii* qPCR: 1 cycle at 95 °C for 10 min followed by 45 cycles of 95 °C for 10 s, 58 °C for 20 s, and 72 °C for 20 s. Based on the positive control sample, intra-assay co-efficient of variation (CV) for the *C. burnetii* qPCR assay was in the range of 0.00–2.06%, and inter-assay CV was 5.9%. Linearity of the *C. burnetii* qPCR assay, PCR efficiency and limit of detection were determined by testing serial dilutions of the positive control sample. The correlation coefficient of the assay was in the range of 0.993–0.998, PCR efficiency was in the range of 100.1–101.36%, and the limit of detection was 2.5 × 10^1^ Genome Equivalents/PCR reaction. As the qPCR target for the *C. burnetii* qPCR (IS1111) is a multi-copy gene, data was not analysed quantitatively; rather, samples were deemed positive for *C. burnetii* only if both duplicates from each sample resulted in a positive qPCR signal. 

#### 2.5.2. Serology

##### *Coxiella burnetii* ELISA

Serum samples were tested for the presence of antibodies against *C. burnetii* using an ELISA (IDEXX Q-Fever antibody test; IDEXX, UK) following the manufacturer’s instructions. A positive and a negative control was supplied by the manufacturer and was included in every assay. The percentage positivity (PP) of each sample was calculated as:

PP = (ODs-ODnc)/(ODpc-ODnc) × 100

where ODs is the optical density (OD) of the sample, ODnc is the average OD of the negative controls and ODpc is the average OD of the positive controls. Sera were considered positive if the PP was equal to or greater than 40%, suspect if the PP was between 30 and 40% and negative if the PP was less than 30%.

##### *Chlamydia abortus* ELISA

Sera collected pre-tupping, at lambing and three weeks post lambing were tested using an in-house *C. abortus* specific rOMP90B-3 indirect ELISA, as described previously [40]. Results were calculated as a percentage positivity, in relation to positive and negative control sera. Sera were considered positive (+) if the OD percentage was greater than 60%, ambiguous (−/+) if the OD percentage was between 50 and 60% and negative (−) if the OD percentage was less than 50%.

##### *Toxoplasma gondii* ELISA

Ovine sera, collected pre-tupping and at lambing, were tested by SRUC Veterinary Services (Midlothian, UK) for reactive *T. gondii* antibodies using a pigtype^®^ Toxoplasma Ab ELISA kit (Indical Biosciences GmbH, Leipzig, Germany). Serum samples with titres above 30% were considered positive (+).

### 2.6. Characterisation of LPS from C. burnetii Phase II Antigen

#### 2.6.1. LPS Extraction

To confirm that the LPS of the phase II vaccine used in this study was truncated, LPS was purified using an LPS Extraction kit (Abcam, Cambridge, UK), according to the manufacturer’s instructions, with the following modifications: the lyophilised phase II antigen (Virion/Serion, Germany) was weighed, and ten volumes of lysis buffer were added to the antigen preparation. Samples were kept in an ice bath and individually sonicated (MISONIX S-3000, Cole-Parmer) three times for 30 s, in a continuous pulse at 2–10 watts and incubated on ice for 10 min. Samples were centrifuged for 10 min at 4 °C at 2500× *g*. The supernatant was transferred to a clean 1.5 mL Eppendorf tube, and 0.1 mg/mL Proteinase K was added. Samples were heated at 60 °C for 60 min and then centrifuged at 4 °C for 10 min at 2500× *g*. Supernatant was transferred to a clean 1.5 mL Eppendorf and stored at −20 °C prior to prior to further analysis.

#### 2.6.2. Visualization of *C. burnetii* LPS

LPS samples extracted from *C. burnetii* phase II antigen were analysed by SDS-PAGE using the NuPAGE^®^ electrophoresis system (Thermo Fisher Scientific, USA). Briefly, samples were prepared in NuPAGE LDS sample buffer with reducing agent and heated to 70 °C for 10 min prior to loading on a NuPAGE™ 4–12% Bis-Tris gel (Thermo Fisher Scientific, USA). Gels were run in MES SDS running buffer and included an LPS standard and a SeeBlue™ Plus2 Pre-Stained Protein Ladder (Thermo Fisher Scientific, USA). Following electrophoresis, in gel LPS was stained using a SilverQuest™ staining kit (Invitrogen, USA) following the manufacturer’s instructions.

### 2.7. Statistical Analysis

To assess the effect of treatment group (1–3) on rectal temperature, linear regression models were fitted to temperature 24 h post first and second vaccinations separately, with baselines temperatures at vaccination and treatment groups used as explanatory variables (no statistically significant differences between treatment groups were observed at the time of vaccination in any case). Post hoc pairwise comparisons between treatment groups were derived from the model fits, with the resulting *p*-values being adjusted for false discovery rate (FDR) using the Benjamini–Hochberg method [41].

The differences in mean antibody response by treatment group (ELISA data expressed as percentage positivity (PP)) were analysed using a generalized additive model (GAM). The model was fitted by the restricted maximum likelihood (REML) method to log10 (PP + 1) as response variable, with identify link function and Gaussian errors. As explanatory terms, the model included treatment group and spline-based smooth terms (one per treatment group) to account for the non-linearity of the relationship of the response with time. Autocorrelation of the measurements on the same animals over time was accounted for by considering a continuous autoregressive correlation structure of order 1 (CAR (1)). Heterogeneous variances by treatment group were allowed. Differences between the linear terms for each treatment group were examined using Wald tests, and the resulting *p*-values were FDR-adjusted.

The real-time PCR-based cumulative positive/negative outcome data were analysed for each type of sample (faecal, milk and vaginal swab) using binomial generalized linear models (GLM) with a logit link function, including treatment group as main effect. Model parameters were estimated by the maximum likelihood method including a bias-reduction correction [42] to accommodate the lack of variation of the outcome within the vaccinated groups.

Finally, the association of treatment group with lamb survival and normal pregnancy was statistically assessed by applying a Fisher’s exact test to the combined treatment groups compared to the control.

All statistical analyses were performed using the R system for statistical computing v3 [43]. Significance tests were assessed at the usual 5% significance level.

## 3. Results

### 3.1. Assessment of Post-Vaccination Reactogenicity

Post vaccination, no notable injection site reactions were observed in any animal. However, a statistically significant effect of treatment group on temperature was detected at 24 h following both the initial vaccination and second vaccination (*p* < 0.001).

Rectal temperatures are shown in Table S1. At 24 h after the first vaccination, the rectal temperatures of ewes in Group 3 (unvaccinated) were statistically significantly lower than either of the two vaccinated groups (*p* < 0.001). The rectal temperature of Group 3 ewes were 0.68 and 0.76 °C lower, on average, than Group 1 and 2, respectively. At 24 h after the second vaccination, the rectal temperatures of ewes in Group 2 (phase II vaccine group) were statistically significantly higher than those in Group 1 (Coxevac^®^ vaccinated) and Group 3 (unvaccinated) (*p* < 0.001). The rectal temperature of Group 2 ewes were 0.79 and 1.00 °C higher, on average, than Group 1 and 3, respectively.

### 3.2. Serological Response to Vaccination and C. burnetii Challenge

**Figure 2:**
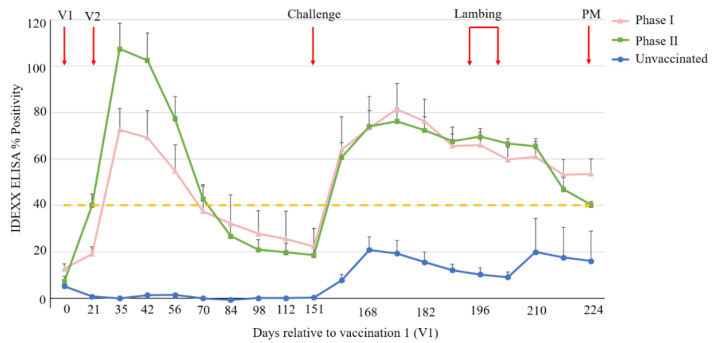
Serological ELISA responses following vaccination and *C. burnetii* challenge. Each point represents the average of each group, with standard error of mean (SEM) displayed. Each group is represented by a unique symbol and colour combination. Ovine sera were considered *C. burnetii* positive if the percentage positivity was ≥ 40% (dashed yellow line). V1 and V2 = vaccination 1 and 2, respectively; Challenge = *C. burnetii* challenge; PM = post-mortem.

For each group, the average serological ELISA response following vaccination and challenge is shown in Figure 2. The *C. burnetii*-specific antibody response in the unvaccinated group (Group 3) was statistically significantly lower across the whole time period compared to either of the two vaccinated groups (*p* < 0.001). Generally, the antibody response, post vaccination and post challenge, did not statistically significantly differ between Groups 1 and 2 (*p* > 0.05), although an examination of fitted trends, Figure S1, suggested a higher antibody response in Group 2 (phase II vaccine group) between day 21 and day 49 (i.e., the four-week period immediately after the second vaccination) compared to Group 1 (Coxevac^®^/phase I vaccine group). According to the 40% positivity threshold of the IDEXX Q-Fever antibody test, at day 35, all ewes in Groups 1 and 2 were seropositive for *C. burnetii*, except for one animal in Group 1 (ewe no. 9704): this ewe seroconverted at low levels following vaccination and antibody levels consistently remained below the positivity threshold. Following a post-vaccination peak at day 35, the average antibody levels in Groups 1 and 2 declined gradually to below the ELISA positivity threshold prior to challenge. Post *C. burnetii* challenge (day 151), average antibody levels in both Groups 1 and 2 increased sharply, peaking on day 175 before decreasing gradually to 53% and 40%, respectively, at the end of the trial (day 224). No seroconversion was observed in Group 3 (unvaccinated) ewes at any point prior to challenge (day 151). Post challenge, seroconversion was observed in five out of the six ewes in Group 3 (ewe no. 9668 did not seroconvert, Figure S2), although all antibody titres were below the ELISA positivity threshold except for one of the five ewes (no. 22164). One animal in Group 3 (no. 23501) exhibited a large increase in serum levels of *C. burnetti* specific antibodies post lambing (day 210), which was not apparent in the other ewes in this group (see Figure S2).

### 3.3. Shedding of C. burnetii in Milk, Faecal and Vaginal Swab Samples

Samples were collected daily from *C. burnetii* challenged ewes on days 0, 1, 2 and 3 post lambing and at weekly time points thereafter until the completion of the trial (~4 weeks post lambing). Groups initially consisted of six ewes each; however, five animals were eventually available in groups 1 and 2, due to abortion in one ewe in Group 1 at 115 days of gestation, due to *C. abortus* infection (see Section 3.6), and the death of one ewe in Group 2, due to complications associated with a vaginal prolapse. Results are summarized in Table 2, Table 3 and Table 4.

#### 3.3.1. *C. burnetii* Shedding on Days 0, 1, 2 and 3, Relative to Lambing

During this time, no *C. burnetii* positive qPCR outcomes were recorded for faecal or milk samples for any of the vaccinated ewes (Groups 1 and 2). None of the vaginal swab samples collected from Group 2 were positive for *C. burnetii*; however, two ewes in Group 1 (no. 9329 and 9914) were qPCR positive at one time-point only. In the non-vaccinated ewes (Group 3), most faecal samples were *C. burnetii* negative except for two ewes (no. 21898 and 23501). Milk and vaginal swab samples from all Group 3 ewes were positive on at least one time-point, with milk and vaginal swab samples from ewe 23501 being qPCR positive at all time-points.

#### 3.3.2. *C. burnetii* Shedding at Weekly Sampling Points

Following the completion of lambing, positive qPCR outcomes over three consecutive weekly sample points and at post-mortem in animals across the three treatment groups were analysed. For faecal samples, the vaccinated groups (Group 1 and 2) had a statistically significantly lower number of positives than the unvaccinated control group (Group 3) (*p* = 0.030). For milk samples, the vaccinated groups (Group 1 and 2) had a statistically significantly lower number of positives than the unvaccinated control group (Group 3) (*p* < 0.001). For vaginal swab samples, the vaccinated groups (Group 1 and 2) had a statistically significantly lower number of positives than the unvaccinated control group (Group 3) (*p* = 0.002). There were no statistically significant differences between the two vaccinated groups for any of the samples tested.

### 3.4. Presence of C. burnetii DNA in Tissue Samples of Ewes and Lambs at Post-Mortem

No *C. burnetii* DNA was detected in any of the collected tissue samples in animals belonging to either of the vaccinated groups (Groups 1 and 2). In the unvaccinated ewes (Group 3), *C. burnetii* DNA was only detected in the SMLN of one ewe (no. 22357) and in the udder, uterus, and placenta (cotyledon and inter-cotyledonary membrane) of another ewe (no. 23501). *C. burnetii* DNA was only detected in the liver of one healthy lamb from an unvaccinated ewe (no. 23501, Group 3).

### 3.5. Lambing Outcomes

The lambing outcomes are shown in Table 5. No abortions attributed to *C. burnetii* challenge were observed during this study. Offspring from ewe no. 9880 and no. 23647 were excluded from the final lambing outcome analysis. Ewe 9880, which aborted triplets, was serologically and qPCR positive for *C. abortus* as detailed in Section 3.6. Ewe 23647 died because of septicaemia subsequent to a vaginal prolapse prior to lambing with no abortifacient agents detected in ewe or lamb tissue samples or in a vaginal swab collected post-mortem.

The effect of the vaccines on the proportions of lambs surviving and of abnormal pregnancies are shown in Table 6 and Table 7, respectively. The analysis of lamb survival showed no statistically significant difference between the vaccinated groups (Group 1 and 2) and the unvaccinated group (*p* = 0.100). A normal pregnancy was defined as all lambs born healthy and which survived to the end of the trial period. A statistically significantly higher number of abnormal pregnancies was noted in the unvaccinated group (Group 3) compared to the vaccinated groups (Group 1 and 2) (*p* = 0.008). There were no significant differences in lambing outcomes between the vaccinated groups (Group 1 and 2).

### 3.6. Chlamydia abortus and Toxoplasma gondii Status

The animals used in this study were previously used in an experimental *C. abortus* vaccine trial. To rule out whether abnormal lambing outcomes observed in this study were associated with *C. abortus* or *T. gondii* infection, serology was performed for both pathogens at multiple time-points: pre-tupping, lambing and at three weeks post lambing (*C. abortus* only), and qPCR performed on placental and lamb tissues at post-mortem. The results are summarized in Tables S2 and S3. All the ewe and lamb samples tested for *T. gondii* were qPCR negative. Pre-tupping 2/6, 1/6 and 3/6 ewes were positive for *T. gondii* antibodies in Groups 1, 2 and 3, respectively, with antibodies levels remaining constant throughout the trial. All ewe samples tested from Group 2 and 3 were qPCR negative for *C. abortus*. Except for one ewe (no. 21996) all ewes from Group 1 were qPCR positive for *C. abortus* in vaginal mucus (day 0) and of these positive animals three were also qPCR positive for placental samples. Pre-tupping 2/6, 4/6 and 2/6 ewes were positive for *C. abortus* antibodies in Groups 1, 2 and 3, respectively. Of these, *C. abortus* antibody levels remained consistent throughout the study apart from one ewe in Group 1 (no. 9880) in which antibody levels increased at each time-point, and one ewe in Group 3 (no. 22357) in which antibody levels increased slightly between the first and second but not the second and third time-points. Ewe no. 9880, which aborted triplets on day 115 of gestation, was PCR positive for *C. abortus* in placental tissues (cotyledon and inter-cotyledonary membrane) and vaginal mucus, collected on the same day, but not for *C. burnetii* or *T. gondii*, indicating that the cause of abortion in this ewe was most likely *C. abortus*. Ewe no. 22357 gave birth to triplets of which one was weak and was euthanized at 24 h. Tissue samples from ewe 22357 and its offspring were PCR negative for *T. gondii* and *C. abortus*, however the SMLN tested positive for *C. burnetii*. Milk, faecal and vaginal swab samples collected from this ewe were positive for *C. burnetii* at 5/8, 1/8 and 5/8 sampling time-points, respectively, indicating that the abnormal lambing outcome was more likely to be associated with *C. burnetii* infection.

### 3.7. Characterisation of C. burnetii Phase II LPS

Phase II *C. burnetii* possess a truncated form of LPS [23]. LPS extracted from the phase II antigen was run on an SDS-PAGE gel, which included an LPS standard and protein ladder, and was then silver stained. For the phase II vaccine, two low-molecular-weight bands of approximately 4 and 7 kDa were present, as indicated in Figure S3, which is consistent with truncated forms of LPS reported by Beare et al. [26].

## 4. Discussion

Vaccination of pregnant ewes with either of the *C. burnetii* study vaccines, phase I (Coxevac^®^) or phase II, induced a strong humoral response that was reactivated post challenge and provided comparable high levels of protection against *C. burnetii* challenge compared to unvaccinated controls. Immunization with either vaccine caused complete cessation of shedding in faecal and milk samples, significantly reduced shedding in vaginal mucus and resulted in a higher proportion of normal pregnancies, compared to unvaccinated controls.

As reported in other ruminant species [29,44] vaccination with Coxevac^®^ caused a statistically significant increase in the mean rectal temperature of ewes in Group 1 at 24 h post vaccination compared to unvaccinated animals (Group 3). Compared to the unvaccinated ewes, the mean rectal temperature of animals in Group 2 (phase II vaccinated) also increased 24 h post initial vaccination and at 24 h after the second vaccination. These results indicate that a phase II vaccine can also cause increases in rectal temperatures, similarly to Coxevac^®^; however, these are expected to be transient. Furthermore, the adjuvant Quil-A has previously been shown to induce a pyrogenic response, which may account for the small, yet statistically significant, increase in the mean rectal temperature observed in Group 2 ewes [45]. The complete lack of injection site reactions in these naïve animals is encouraging for the development of a phase II *C. burnetii* vaccine, although testing in animals previously exposed to *C. burneiti* is required to fully assess the potential for reactogenicity.

The results observed in sheep inoculated with the phase I vaccine Coxevac^®^, particularly reductions in shedding following vaccination, are similar to those observed in several goat vaccination studies [30,31,46]. In Europe, Coxevac^®^ is currently licensed for use in cattle and goats [44]; however, this study illustrates that high levels of protection are also afforded in sheep. Arricau-Bouvery et al. [31] examined the impact of phase I and phase II *C. burnetii* vaccines in goats, reporting high levels of protection in goats vaccinated with Coxevac^®^; however, the phase II vaccine used was found to be completely ineffectual [31]. The same authors [31] used the Chlamyvax FQ phase II inactivated vaccine (Merial, France) [47]; however, production of this vaccine has since terminated, and an alternative phase II vaccine formulation was used in the current study that may account for the differences observed. One major difference is that the phase II vaccine used in the current study was adjuvanted with the saponin-based adjuvant Quil-A, whereas Chlamyvax FQ used an oil emulsion adjuvant system. As saponins are known to effectively induce cell-mediated immune (CMI) responses to inactivated antigens [48] and CMI responses have been shown in mice to be required for effective clearance of *C. burnetii* [49], it may be that the protection observed with the phase II study vaccine was due to more effective CMI induction compared to Chlamyvax FQ. Alternatively, the double LPS band observed in the phase II LPS extract may indicate that the phase II antigen is of intermediate virulence, comparable to the NMC strain.

Moos and Hackstadt [21] reported that the pyrogenic response and seroconversion induced by the NMC strain in guinea pigs was analogous to that of animals infected with phase I organisms, although the strain was unable to persist in vivo [22]. These results are similar to this study whereby immunization with either vaccine, phase I or phase II, induced a strong, homologous, serological response in ewes. Previously, the truncated LPS of the NMC variant, which displayed no serological reactivity to phase I, was recovered from the Australian QD strain of *C. burnetii* following 177 passages in eggs [21]. The Nine-Mile phase II antigen used in this study was prepared following 166 passages in embryonated hen’s eggs, and it is possible that a mutant with intermediate virulence and a novel LPS profile may have arisen during production.

Beare et al. [26] examined LPS phase transition in multiple phase I strains at passage 2, 10, 20 and 30, noting that each contained a unique LPS profile at passage 2, similar to previous reports [21,23,26,50]. Beare et al. [26] reported apparent reversion to an intermediate form of LPS following 30 passages of the phase I strain S Q217, accompanied by a decrease in phase II LPS [26]. Furthermore, following successive passage, an upper phase II LPS (~6 kDa), not recognized by anti-phase II antibodies, was observed in the Nine-Mile (RSA363) and Dugway (7E65-68) strains alongside the lower phase II LPS (~3 kDa) [26]. Another study also noted this upper phase II form in the Priscilla strain, which was still present following 90 passages [51]. Extraction of LPS from the phase II antigen used in this investigation revealed a double LPS band exhibiting a similar upper and lower band profile. These upper LPS bands may represent an alternate intermediate LPS, facilitating recognition of *C. burnetii* by pathogen recognition receptors on host dendritic cells, thus inducing a protective immune response.

Additionally, or alternatively, physiological and/or immunological differences between the ovine and caprine species [52,53] may also contribute towards these disparate results. Studies have shown that sheep are less susceptible to the pathogenic effects of *C. burnetii* infection than goats, with a higher seroprevalence generally reported in the latter [7,54]. Furthermore, abortions and production of weak offspring are more common in goats, with sheep less likely to display clinical signs of Q-fever [2,55]. A major difference between the *C. burneiti* ovine and caprine models appear to be the serological response of unvaccinated animals to challenge. Arricau-Bouvery et al. [31] reported there was a large and sustained increase in *C. burnetii* specific antibodies in unvaccinated goats post challenge; however, in the current study, the increase in specific antibody titres in unvaccinated sheep post challenge was marginal and generally began to decline towards the end of the trial. These discrete responses could indicate differences in innate immunity between these two ruminant species following *C. burnetii* infection, which may facilitate earlier control and/or clearance of the bacteria in sheep compared to goats, explaining the dampened humoral response observed in unvaccinated animals in this study. A comparison of the challenge dose between this study and that of the goat study conducted by Arricau-Bouvery et al. [31] further exemplifies differences in susceptibility to *C. burnetii* between these two species. In the current experimental trial, ewes were challenged with an IMD of 10^6^, whereas in the goat study animals were given an IMD of 10^4^ *C. burnetii* [31]. Despite sheep in this investigation receiving 100-fold more bacteria, no abortions due to *C. burnetti* were observed, and ewes did not display any clinical symptoms of Q-fever, in contrast to infected goats [31].

The ewes utilized during the current study were also tested for *C. abortus* and *T. gondii*, common ovine abortifacient agents. The serological results for *T. gondii* indicated that at least one ewe from every group had previously been exposed to the parasite, although the stability of the ELISA results over time, and the negative tissue qPCR results suggested the study animals were not harbouring an active *T. gondii* infection. As animals were recruited from a *C. abortus* vaccine study, it was unsurprising that multiple ewes from each group were serologically positive for *C. abortus*; however, evidence of an active chlamydial infection was only apparent for one ewe in Group 1 (no. 9880). This animal aborted triplets, approximately 4 weeks prior to lambing, and was qPCR positive for *C. abortus* in the placental tissues and vaginal mucus. Ewe no. 22357 exhibited a small increase in *C. abortus* antibody titres; however, the causative agent of the weak lamb borne to this animal was judged to be *C. burnetii*. *C. abortus* was not detected in placental tissue or vaginal mucus of this ewe, whereas *C. burnetii* was present in the SMLN and in milk and vaginal mucus at multiple sampling time points.

The results of this study and others highlight that, even in the absence of abortion, *C. burnetii* should still be considered as a threat to sheep due to potential increases in abnormal pregnancies [2]. Interestingly, one ewe in Group 3 (no. 23501, unvaccinated), which seroconverted at low levels post challenge, exhibited a strong reactivation of *C. burnetii* specific antibodies post lambing (day 210). Ewe number 23501 was the only unvaccinated animal to consistently shed *C. burnetii* in milk and vaginal mucus samples and was the only unvaccinated ewe to give birth to offspring that were all registered as healthy. Ewe 23501 exhibited high levels of bacterial shedding and displayed the highest antibody response of all unvaccinated animals indicating a possible positive correlation between bacterial load and serology. Furthermore, the only lamb in this study in which *C. burnetii* DNA was detected was the progeny of ewe 23501. Studies have shown that ruminants infected with *C. burnetii* can produce healthy offspring or may even give birth to both healthy and dead young, as observed in this study, indicating that infection does not necessarily lead to negative pregnancy outcomes [56]. It is noteworthy that multiple human Q-fever outbreaks attributed to sheep have arisen in cases where no ovine abortions were reported [57,58]. Roest et al. [56] reported that *C. burnetii* was initially detected in the organs of kids, from infected goats, but at day 28 post parturition, the bacterium was undetectable. In the current study any surviving lambs were culled between 23 and 29 days post parturition, and these later sampling time-points may explain why *C. burnetii* DNA was only identified in the liver of one lamb; future studies may benefit from earlier sampling points. Ewe serum samples from all three groups were tested for bacteraemia and were consistently negative throughout the trial, similarly to results reported in *C. burnetii* infected goats [56].

Few studies have examined protection elicited by the phase I vaccine Coxevac^®^ in sheep; however, Brooks et al. [33] carried out a similar study in which pregnant ewes were vaccinated with either a formalin-inactivated whole-cell phase I Henzerling strain vaccine or a chloroform methanol residue vaccine of the Nine-Mile strain. After challenge, both phase I vaccines provided protection and reduced, but did not completely eliminate, environmental shedding, similarly to results reported in the current study.

Serological *C. burnetii* ELISA testing is often used for diagnosis and monitoring of ruminants. The results from the current study indicate that infected animals may seroconvert at low levels, or not at all, despite shedding bacteria in faeces, milk and vaginal mucus. This lack of correlation between seropositivity and shedding has been observed previously in ovine, bovine and caprine studies [10,46,59,60]. The results of the current study and those of others highlight that serology alone is not a comprehensive tool for detection of Q-fever in individual sheep. Furthermore, surveillance of *C. burnetii* in ruminants could be hampered by temporal shedding patterns exhibited in milk, faecal and vaginal mucus samples [10,46]. Except for one ewe (no. 23501) in Group 3, all non-vaccinated/challenged ewes exhibited transient shedding of the bacterium in milk, faeces and vaginal swab samples. A requirement for multiple time-point sample collection and possible disassociation between serology and shedding underscores the need for improved Q-fever detection and control methods.

The cost and safety issues, both for humans and animals, associated with the production and/or use of phase I *C. burnetii* vaccines necessitate the development of a novel, safe and effective Q-fever vaccine. The findings presented here, exhibiting comparable results for phase I and phase II vaccines in a pregnant ewe challenge model, highlight that a phase II vaccine formulation may be an appropriate alternative. Further investigation including repetition of this vaccine/challenge trial in a pregnant goat model would be extremely useful to validate the utility of these results and assess how they may be applied in the wider context in the control of Q-fever. Naturally, both humans and animals usually become infected with *C. burnetii* following inhalation of contaminated spores [11]. The animals in this study were experimentally infected via the subcutaneous route, an unnatural method of infection, which may have impacted results. Roest et al. [56] examined the impact that three different routes of inoculation had on experimentally infected goats. Intranasal inoculation resulted in increased bacterial loads in the placenta and caused abortions at earlier time-points compared to subcutaneous or oral inoculation routes [56]. In sheep the influence of infection route upon dissemination and excretion of the bacterium has not been investigated. As with goats, the intranasal route may improve bacterial colonization and investigation of this technique in sheep combined with the vaccines utilized in this study would more closely reflect natural conditions.

## 5. Conclusions

Both study vaccines, phase I and phase II, induced a strong humoral response in sheep and demonstrated protection again *C. burnetii*, as measured by reduced bacterial shedding and production of healthy lambs. Previously, Q-fever was identified as an occupational hazard, restricted to those working closely with ruminants. The unanticipated Dutch outbreak has highlighted the threat of *C. burnetii* to public health and underscores the need for improved control strategies. This study demonstrated that the commercially available phase I vaccine Coxevac^®^ is highly efficacious in sheep and implementation into ovine vaccination programs could help to reduce human Q-fever cases. Additionally, it is proposed that the phase II vaccine used in this trial may offer a safer and more cost-effective alternative to the current Q-fever vaccines.

## Figures and Tables

**Table 1:** Sequences and concentrations of oligonucleotide primers and probes used in this study.

Pathogen	Primer/Probe	Nucleotide Sequence 5′-3′	Concentration (nM)	Ref
*C. burnetii*	IS1111-F	catgacattgccgcgtttac	400	
	IS1111-R	ggttggtccctcgacaacat	400	[36]
	IS1111-probe	FAM-aatccccaacaacacctccttattcccac-BHQ-1	200	
*C. abortus*	MOMP-F	gcggcattcaacctcgtt	300	
	MOMP-R	ccttgagtgatgcctacattgg	300	[38]
	MOMP-probe	FAM-tgttaaaggatcctccatagcagctgatcag-TAMRA	250	
*T. gondii*	TOX-F	aggagagatatcaggactgtag	700	
	TOX-R	gcgtcgtctcgtctagatcg	700	
	TOX-probe	FAM-ccggcttggctgcttttcct-BHQ-1	250	[39]
	CIAC-probe	JOE-agcgtaccaacaagtaattctgtatcgatg-BHQ-1	200	

F = Forward primer, R = Reverse primer, FAM = Fluorescein amidites, BHQ = Black Hole Quencher, TAMRA = Tetramethylrhodamine, JOE = 5′-Dichloro-dimethoxy-fluorescein.

**Table 2:** Presence (+) or absence (-) of *C. burnetii* DNA in milk samples. Samples were collected on day of lambing (0) and then for three consecutive days (1, 2 and 3). Samples were then collected weekly for 3 weeks, with a final sample set collected immediately prior to post-mortem (PM).

		Milk Samples							
**Vaccine Group**	**Ewe** **No.**	**Days Post Lambing**				**Weekly Sample Points**			**PM**
		0	1	2	3	WK 1	WK 2	WK 3	
Group 1: Coxevac^®^ vaccinated	9329	-	-	-	-	-	-	-	-
	9360	-	-	-	-	-	-	-	-
	9914	-	-	-	-	-	-	-	-
	21996	-	-	-	-	-	-	-	-
	22155	-	-	-	-	-	-	-	-
Group 2: Phase II vaccinated	9315	-	-	-	- *		-	-	-
	9612	-	-	-	-	-	-	-	-
	9888	-	-	-	-	-	-	-	-
	9902	-	-	-	-	-	-	-	-
	23161	-	-	-	-	-	-	-	-
Group 3: Unvaccinated controls	9668	-	+	+	-	+	+	+	+
	21898	-	+	+	+	+	+	+	-
	22164	-	-	-	+	+	-	+	+
	23501	+	+	+	+	+	+	+	+
	23538	-	-	+	-	-	-	+	+
	22357	-	-	-	+	+	+	+	+

* 3 days post lambing and week 1 sampling time-points fell on the same day for this ewe, indicated by the boxed result.

**Table 3:** Presence (+) or absence (-) of *C. burnetii* DNA in vaginal swab samples. Samples were collected on day of lambing (0) and then for three consecutive days (1, 2 and 3). Samples were then collected weekly for 3 weeks, with a final sample set collected immediately prior to post-mortem (PM).

		Vaginal Swab Samples							
**Vaccine** **Group**	**Ewe** **No.**	**Days Post Lambing**				**Weekly Sample Points**			**PM**
		0	1	2	3	WK 1	WK 2	WK 3	
Group 1: Coxevac^®^ vaccinated	9329	-	-	-	+	-	-	-	-
	9360	-	-	-	-	-	-	-	-
	9914	-	+	-	-	-	-	-	-
	21996	-	-	-	-	-	-	-	-
	22155	-	-	-	-	-	-	-	-
Group 2: Phase II vaccinated	9315	-	-	-	- *		-	-	-
	9612	-	-	-	-	-	-	-	-
	9888	-	-	-	-	-	-	-	-
	9902	-	-	-	-	-	-	-	-
	23161	-	-	-	-	-	-	-	-
Group 3: Unvaccinated controls	9668	-	+	+	+	-	+	+	+
	21898	-	-	+	+	-	-	+	+
	22164	-	-	+	-	-	-	+	+
	23501	+	+	+	+	+	+	+	+
	23538	-	-	-	+	+	+	-	+
	22357	-	-	+	-	+	+	+	+

* 3 days post-lambing and week 1 sampling time-points fell on the same day for this ewe, indicated by the boxed result.

**Table 4:** Presence (+) or absence (-) of *C. burnetii* DNA in faecal samples. Samples were collected on day of lambing (0) and then for three consecutive days (1, 2 and 3). Samples were then collected weekly for 3 weeks, with a final sample set collected immediately prior to post-mortem (PM).

		Faecal Samples							
**Vaccine Group**	**Ewe** **No.**	**Days Post-Lambing**				**Weekly Sample Points**			**PM**
		0	1	2	3	WK 1	WK 2	WK 3	
Group 1: Coxevac^®^ vaccinated	9329	-	-	-	-	-	-	-	-
	9360	-	-	-	-	-	-	-	-
	9914	-	-	-	-	-	-	-	-
	21996	-	-	NA	-	-	-	-	-
	22155	-	-	-	-	-	-	-	-
Group 2: Phase II vaccinated	9315	-	-	-	-*		-	-	-
	9612	NA	-	-	-	-	-	-	-
	9888	-	-	-	NA	-	-	-	-
	9902	NA	-	-	-	-	-	-	-
	23161	-	-	-	-	-	-	-	-
Group 3: Unvaccinated controls	9668	-	-	-	NA	-	+	+	-
	21898	-	NA	-	+	-	+	+	-
	22164	-	-	-	NA	-	-	+	-
	23501	+	+	+	+	+	+	-	-
	23538	-	-	-	-	-	+	-	-
	22357	-	-	-	-	+	-	-	-

* 3 days post lambing and week 1 sampling fell on the same day, indicated by the boxed result. NA = sample not available.

**Table 5:** Lambing outcome results.

Group	Ewe No.	Parity	Healthy Lambs	Stillborn	Neonatal Death	Aborted	Weak Lambs
1: Coxevac® vaccinated	9329	Twins	2	0	0	0	0
	9360	Twins	2	0	0	0	0
	9914 ^a^	Triplets	2	0	1@24hrs	0	0
	21996	Twins	2	0	0	0	0
	22155	Twins	2	0	0	0	0
	9880	Triplets	0	0	0	3 ^b^	0
2: Phase II vaccinated	9315	Twins	2	0	0	0	0
	9612	Twins	2	0	0	0	0
	9888	Twins	2	0	0	0	0
	9902	Twins	2	0	0	0	0
	23647	Twins ^c^	0	0	0	0	0
	23161	Triplets	3	0	0	0	0
3: Unvaccinated controls	9668	Twins	1	0	0	0	1
	21898	Twins	1	1	0	0	0
	22164	Twins	1	0	1@72hrs	0	0
	23501	Twins	2	0	0	0	0
	23538	Twins	1	1	0	0	0
	22357	Triplets	2	0	0	0	1E@24hrs

^a^ Scanned as twins, ^b^ *C. burnetii* negative; *C. abortus* positive, ^c^ Ewe died pre-lambing, E = euthanized. Ewe numbers in bold were excluded from the final analysis.

**Table 6:** Number of lambs that either survived or died following parturition.

Group	Survived	Died	Total
1: Coxevac^®^ vaccinated	10	1	11
2: Phase II vaccinated	11	0	11
3: Unvaccinated controls	8	4	12

**Table 7:** Number of normal or abnormal pregnancies.

Group	Normal ^a^	Abnormal ^b^	Total
1: Coxevac^®^ vaccinated	4	1	5
2: Phase II vaccinated	5	0	5
3: Unvaccinated controls	1	5	6

^a^ Normal pregnancy, defined as all lambs born healthy and survived, ^b^ Abnormal pregnancies attributed to *C. burnetii* infection.

## Data Availability

The data presented in this study are available on request from the corresponding author.

## Supplementary Materials

The following supporting information can be downloaded at: https://www.mdpi.com/article/10.3390/vaccines11030511/s1, Figure S1. Plot of individual antibody response values alongside the trends fitted by the generalized additive model for each group; Figure S2. Serological ELISA responses of challenged ewes only, following vaccination and *C. burnetii* challenge; Figure S3. Phase II *C. burnetii* antigen LPS extract; Table S1: Rectal temperatures of ewes at primary and secondary vaccination; Table S2: Presence or absence of *T. gondii* and *C. abortus* antibodies present in ewe serum samples at pre-tupping, lambing and for *C. abortus* only at 3 weeks post lambing; Table S3. Presence or absence of *C. abortus* DNA in milk vaginal swab (VS) samples on day 0 of lambing and in placenta samples, inter-cotyledonary membrane (A) and cotyledon (B).
