# Supplementary material for: Efficacy of Phase I and Phase II Coxiella burnetii Bacterin Vaccines in a Pregnant Ewe Challenge Model
Source: Vaccines (Basel). 2023 Feb 22;11(3):511. doi: 10.3390/vaccines11030511 (PMC10054861; doi:10.3390/vaccines11030511)

Figure S1. Plot of individual antibody response values alongside the trends fitted by the generalized additive model for each group. Each group is represented by a unique colour combination. The time is shown in weeks since the first vaccination. Each point represents the antibody response of an individual animal and the shaded areas represent the 95% confidence intervals. The antibody responses in the unvaccinated group were significantly lower across the whole trial than the responses in either vaccinated group ($p < 0.001$). Generally, there is no evidence of a consistent significant difference across the whole time-period between the vaccinated groups ($p > 0.05$). However, in the time period between approximately two to five weeks the 95 % confidence intervals for group 1 and 2 do not overlap providing evidence to support a higher antibody response in animals which received the phase II vaccine compared to those that received the phase I vaccine. The antibody levels in group 1 and 2 appear to be equivalent at all other time-points.

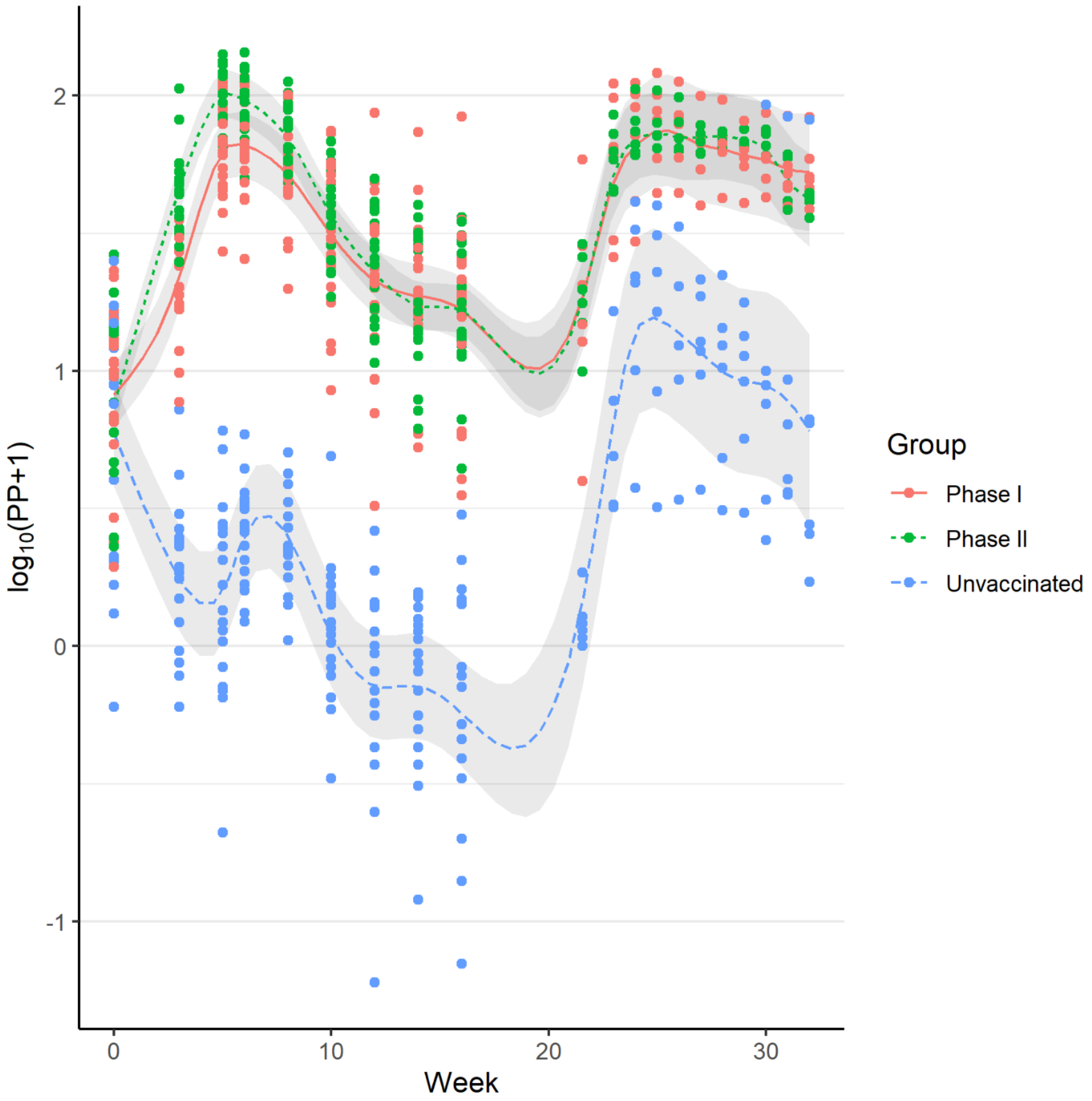

Supplement: Supplementary file 1 [file vaccines-11-00511-s001.zip › Figure S1.pdf]
