# Supplementary material for: Efficacy of Phase I and Phase II Coxiella burnetii Bacterin Vaccines in a Pregnant Ewe Challenge Model
Source: Vaccines (Basel). 2023 Feb 22;11(3):511. doi: 10.3390/vaccines11030511 (PMC10054861; doi:10.3390/vaccines11030511)

Figure S2. **Serological ELISA responses of challenged ewes only, following vaccination and *C. burnetti* challenge**. Each point represents the response of an individual ewe. In each graph individual animals are represented by a unique colour. (A) Phase I (Coxevac®), (B) Phase II, (C) Unvaccinated. Ovine sera were considered *C. burnetii* positive if the percentage positivity was ≥ 40 % (dashed yellow line). V1 and V2 = vaccination 1 and 2, respectively; C = *C. burnetii* challenge; PM = post-mortem.

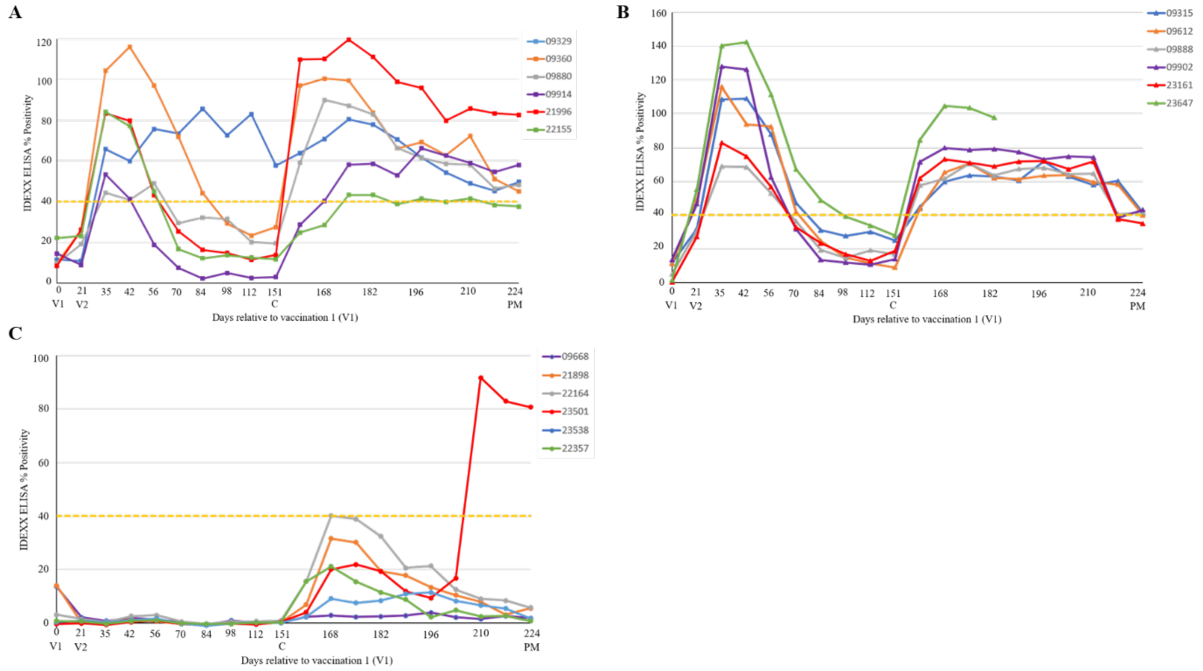

Supplement: Supplementary file 1 [file vaccines-11-00511-s001.zip › Figure S2.pdf]
