# Supplementary material for: Efficacy of Phase I and Phase II Coxiella burnetii Bacterin Vaccines in a Pregnant Ewe Challenge Model
Source: Vaccines (Basel). 2023 Feb 22;11(3):511. doi: 10.3390/vaccines11030511 (PMC10054861; doi:10.3390/vaccines11030511)

Figure S3. Phase II *C. burnetii* antigen LPS extract. SDS-PAGE gel of LPS extracted from the phase II *C. burnetii* antigen preparation used in the phase II vaccine. (1) LPS standard, (2) Phase II LPS, (L) SeeBlue Plus2 protein ladder (molecular weights in kDa are indicated on the right hand side of the gel image). Upper and lower phase II LPS bands are visible in lane 2.

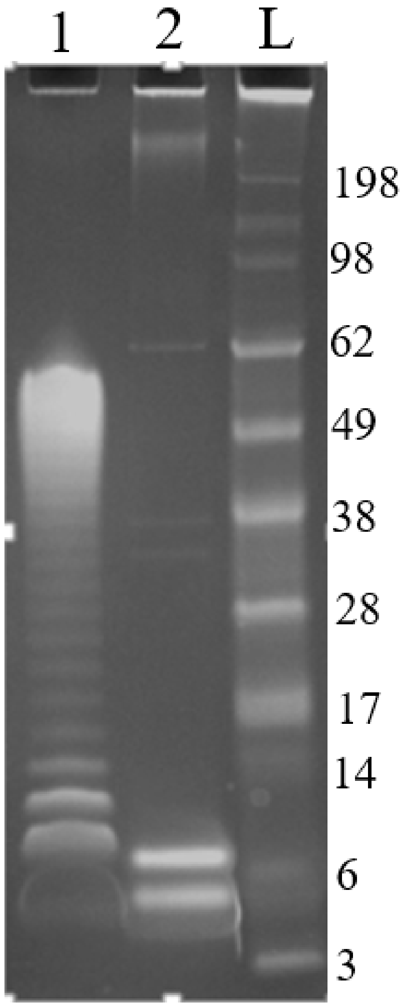

Supplement: Supplementary file 1 [file vaccines-11-00511-s001.zip › Figure S3.pdf]
