# Supplementary material for: Efficacy of Phase I and Phase II Coxiella burnetii Bacterin Vaccines in a Pregnant Ewe Challenge Model
Source: Vaccines (Basel). 2023 Feb 22;11(3):511. doi: 10.3390/vaccines11030511 (PMC10054861; doi:10.3390/vaccines11030511)
Supplement: Supplementary file 1 [file vaccines-11-00511-s001.zip › Table S1.pdf]

Table S1. Rectal temperatures of ewes at first and second vaccinations. Temperatures were recorded at the time of each vaccination and 24 hrs post-vaccination.

Group	Ewe No.	First vaccination Temp. (°C)		Second vaccination Temp. (°C)	
		0 hr	24 hr	0 hr	24 hr
1: Coxevac® vaccinated	5043	38.2	40.8	39.40	39.3
	8539	40.6	40.3	39.90	40.2
	8625	39.7	41.0	39.70	39.5
	**9329**	39.9	40.1	39.80	39.8
	**9360**	39.4	39.9	39.40	39.3
	9661	40.2	40.9	39.2	39.5
	9704	39.7	41.0	39.70	39.5
	9872	40.6	40.5	40.50	40.2
	**9880**	39.8	40.1	39.60	39.4
	**9914**	40.1	39.7	39.50	39.4
	9930	40.4	40.8	39.80	40.2
	9938	39.9	40.3	39.70	39.6
	10736	40.1	41.2	39.80	40.3
	**21996**	39.6	40.7	39.50	39.3
	22026	40.0	40.6	39.80	39.5
	**22155**	40.2	40.3	39.80	39.3
	22246	40.2	40.3	39.50	40.0
	22275	39.9	39.8	39.70	39.8
	22325	39.6	41.0	39.80	39.2
	23502	40.2	40.7	39.90	39.7
2: Phase II vaccinated	04408	40.1	40.6	39.5	39.7
	08927	39.8	40.6	39.8	40.12
	09306	40.1	40.6	39.6	42.2
	**09315**	39.7	40.9	39.3	41.1
	**09612**	40	40.4	39.9	40.5
	09665	39.4	40.2	39.3	40.1
	09674	39.7	40.8	40.1	40.6
	09873	39.7	40.6	39.5	40.7
	**09888**	39.7	40.2	39.3	39.8
	**09902**	39.8	41.1	39.7	40.3
	09936	39.6	40.4	39.3	40.5
	09948	39.6	40.3	39.8	40.4
	22112	39.9	40.6	39.8	40.6
	22159	39.3	40.5	39.4	40.4
	22168	39.7	40.7	39.4	40.5
	22282	40.1	40.4	40	40.1
	22354	40.5	40.6	40	40.3
	23161	39.6	40.4	39.4	40.2
	**23647**	40	40.6	39.5	39.9
	**23681**	40.2	40.9	40	40.8

Table S1 continued:

Group	Ewe No.	First vaccination Temp. (°C)		Second vaccination Temp. (°C)	
		0 hr	24 hr	0 hr	24 hr
3:Unvaccinated control	4213	39.6	39.7	39.1	39.0
	8917	40.3	40.1	39.7	39.6
	9313	39.5	39.6	39.4	39.1
	9328	39.8	40.1	39.8	39.6
	9338	40.0	40.1	40.0	39.7
	**9668**	40.3	40.4	39.9	39.4
	9698	39.7	39.7	39.5	39.3
	9861	40.0	39.8	39.7	39.2
	9899	40.2	39.7	39.7	38.9
	9912	39.7	39.8	39.7	39.3
	9928	40.0	39.5	39.5	39.4
	10699	40.2	40.1	39.7	39.7
	**21898**	39.9	39.9	39.8	39.4
	22000	39.9	39.8	39.8	39.5
	**22164**	39.6	39.8	40.1	39.7
	22228	39.8	39.2	40.4	40.7
	22271	39.6	39.5	39.4	39.1
	**23501**	40.2	39.6	39.5	39.2
	**23538**	39.7	39.8	39.7	39.1
	**22357**	39.4	40.1	40.0	39.9

Temp. (°C) = rectal temperature. Bold numbers indicate ewes selected for *C. burnetii* challenge
