# Supplementary material for: Efficacy of Phase I and Phase II Coxiella burnetii Bacterin Vaccines in a Pregnant Ewe Challenge Model
Source: Vaccines (Basel). 2023 Feb 22;11(3):511. doi: 10.3390/vaccines11030511 (PMC10054861; doi:10.3390/vaccines11030511)
Supplement: Supplementary file 1 [file vaccines-11-00511-s001.zip › Table S2.pdf]

Table S2. Presence (+) or absence (-) of *T. gondii* and *C. abortus* antibodies present in ewe serum samples at pre-tupping, lambing and for *C. abortus* only at 3 weeks post lambing.

Group	Ewe No.	*T. gondii* ELISA Results		*C. abortus* ELISA Results		
		Pre-tupping	Lambing	Pre-tupping	Lambing	3 weeks post lambing
1: Coxevac® vaccinated	9329	-	-	-	-	-
	9360	-	-	-	-	-
	9914	-	-	-	-	-
	21996	+	+	-	-	-
	22155	+	+	+	+	+
	9880	-	-	+	+	+
2: Phase II vaccinated	9315	-	-	+	+	+
	9612	-	-	+	+	-/+
	9888	-	-	-	-	-
	9902	-	-	+	+	-/+
	23647	-	N/A*	-	N/A*	N/A*
	23161	+	+	+	-/+	-/+
3:Unvaccinated control	9668	-	-	-	-	-
	21898	+	+	+	+	+
	22164	+	+	-	-	-
	23501	+	+	-	-	-
	23538	-	-	-	-	-
	22357	-	-	+	+	+

*N/A = sample not available
