# Supplementary material for: Efficacy of Phase I and Phase II Coxiella burnetii Bacterin Vaccines in a Pregnant Ewe Challenge Model
Source: Vaccines (Basel). 2023 Feb 22;11(3):511. doi: 10.3390/vaccines11030511 (PMC10054861; doi:10.3390/vaccines11030511)
Supplement: Supplementary file 1 [file vaccines-11-00511-s001.zip › Table S3.pdf]

Table S3. Presence (+) or absence (-) of *C. abortus* DNA in milk vaginal swab (VS) samples on day 0 of lambing and in placenta samples, inter-cotyledonary membrane (A) and cotyledon (B).

Group	Ewe No.	*C. abortus* PCR Results		
		VS Day 0	Placenta (A)	Placenta (B)
1: Coxevac® vaccinated	9329	+	-	-
	9360	+	+	-
	9914	+	+	-
	21996	-	-	-
	22155	+	-	-
	9880	+	+	+
2: Phase II vaccinated	9315	-	-	-
	9612	-	-	-
	9888	-	-	-
	9902	-	-	-
	23647	-	-	-
	23161	-	-	-
3: Unvaccinated control	9668	-	-	-
	21898	-	-	-
	22164	-	-	-
	23501	-	-	-
	23538	-	-	-
	22357	-	-	-
